# The relationship between physical activity trajectories and frailty: a 20-year prospective cohort among community-dwelling older people

**DOI:** 10.1186/s12877-022-03493-7

**Published:** 2022-11-16

**Authors:** Yen-Kuang Lin, Chen-Yueh Chen, Denise Shuk Ting Cheung, Jed Montayre, Chen-Yin Lee, Mu-Hsing Ho

**Affiliations:** 1 Graduate Institute of Athletics and Coaching Science, National Taiwan Sport University, No. 250, Wenhua 1st Rd, 33301 Guishan, Taoyuan Taiwan; 2 Doctoral Program of Transnational Sport Management and Innovation, National Taiwan Sport University, No. 250, Wenhua 1st Rd, 33301 Guishan, Taoyuan Taiwan; 3 School of Nursing, LKS Faculty of Medicine, The University of Hong Kong, 5/F, Academic Building, 3 Sassoon Road, Pokfulam, Hong Kong Hong Kong; 4 School of Nursing and Midwifery, Western Sydney University, Sydney, 2560 NSW Australia; 5 Graduate Institute of Educational Art and Healing, Mingdao University, 369 Wen-Hua Rd, 52345 Pitou, Changhua, Taiwan

**Keywords:** Group-based trajectories, Physiological change, Predictor, primary health care

## Abstract

**Background:**

Studies on examining the relationship between physical activity patterns and frailty are lacking. This study examined physical activity patterns in older people and investigated the relationship between physical activity and frailty as well as identifying the predictors of frailty.

**Methods:**

We used a nationally representative longitudinal database, the Taiwan Longitudinal Study of Aging (TLSA) database, and data for a 20-year period were extracted and analyzed. A total of 5131 participants aged ≥ 60 years in 1996 were included in the current analysis. Information regarding demographic characteristics, frailty, physical activity, comorbidities, oral health, and depressive symptoms was extracted from the TLSA database. Physical activity patterns were examined using group-based trajectory modeling from 1996 to 2015. Potential predictors were examined by performing multivariate logistic regression.

**Results:**

Four trajectories of the physical activity pattern were found: consistently physically inactive (33.7%), consistently physically active (21.5%), incline (21.6%), and decline (23.2%). Throughout the period, the trajectories of the four groups significantly differed from each other at year 2015, with the incline and decline groups exhibiting the lowest and highest frailty scores, respectively (*p* < 0.001). Older age, male, poor oral health, diabetes, chronic kidney disease, and depressive symptoms were identified as risk factors for frailty.

**Conclusion:**

Physical activity reduces the risk of chronic conditions, which contributes to healthy longevity. This study can guide the development of future research and interventions to manage frailty in older people, particularly in considering previous physical activity trajectories within the life course.

## Background

With longevity observed among populations, health problems such as chronic diseases in later life have become more common worldwide. In Taiwan, the proportion of adults aged ≥ 65 years was 11% in 2010, and by 2025, Taiwan is expected to become a super-ageing society, with 20% of its population being aged > 65 years [1]. Recently, many researchers have been focusing on age-related problems such as frailty, which is a commonly observed syndrome in older adults. Frailty is a medically distinct syndrome characterized by multi-system physiological changes [2]. Frailty does not explicitly imply disease status. Although some older people develop frailty without any life-threatening illness, frailty is strongly correlated with an increased risk of severe health outcomes such as falls, hospitalization, and mortality [3]. Frailty is considered as one of the most significant health concerns among older populations because it results from a cumulative decline in the physiological system depleting homoeostatic reserves, where even minor stressor may trigger disproportionate changes in health status [4]. The prevalence of frailty in the community ranged from 4.0 to 59.1% [5]. A recent study examining 1014 Taiwanese participants estimated the prevalence of frailty and prefrailty to be 17.6% and 23.1%, respectively [6]. Because frailty is a high-priority and challenging health problem in the aging population, identification of modifiable risk factors is crucial for administering early intervention for preventing or delaying the incidence of frailty.

Physical inactivity is one of the most common risk factors associated with frailty in older people. In cross-sectional [7], prospective longitudinal [8], and case–control experimental studies [9], the physically active individuals tend to have higher physical fitness, which is also a protective factor against various chronic diseases including hypertension [10], diabetes mellitus (DM) [11], osteoporosis [12], cancer [13], and depression [13]. An epidemiological study demonstrated that the risk of all-cause mortality was higher in physically inactive people [14]. Physical activity exerts positive effects on mood and physical function. Moreover, better physical well-being may lead to improved psychological well-being. However, the findings of some intervention studies on single physical activity intervention were unsatisfactory [15], which warrant the need for strong evidence demonstrating the effects of physical activity interventions on frailty. The association of different physical activity patterns with frailty and whether physical activity can be used for the treatment of frailty remains unclear. Studies have examined the effect of physical activity on the risk of falls, which is an adverse outcome of frailty. Baker et al. (2007) demonstrated that a combination of aerobic, balance, and physical activity training can help reduce fall risk [16]. Mobility function is another major concern for frailty. Latham et al. found limited effectiveness of resistance physical activity training in reducing the risk of disability in the activities of daily living (ADL) in the older population [17].

To sum up, the findings of the aforementioned studies have indicated the positive effects of physical activity on older adults who are frail or have a high risk of frailty. Several areas related to physical activity as an intervention still require further investigation. Before recommending regular physical activity for older adults to address frailty and its related adverse outcomes, stronger evidence is required in terms of physical activity patterns. As a result, our research aims are threefold: Firstly, to identify various physical activity pattern in older adults. Secondly, to explore the potential risk factors for frailty. Lastly, to investigate the hypothesis that whether the trajectory patterns of physical activity are correlated with long-term frailty outcome.

## Materials and methods

### Study design and participants

**Fig. 1:**
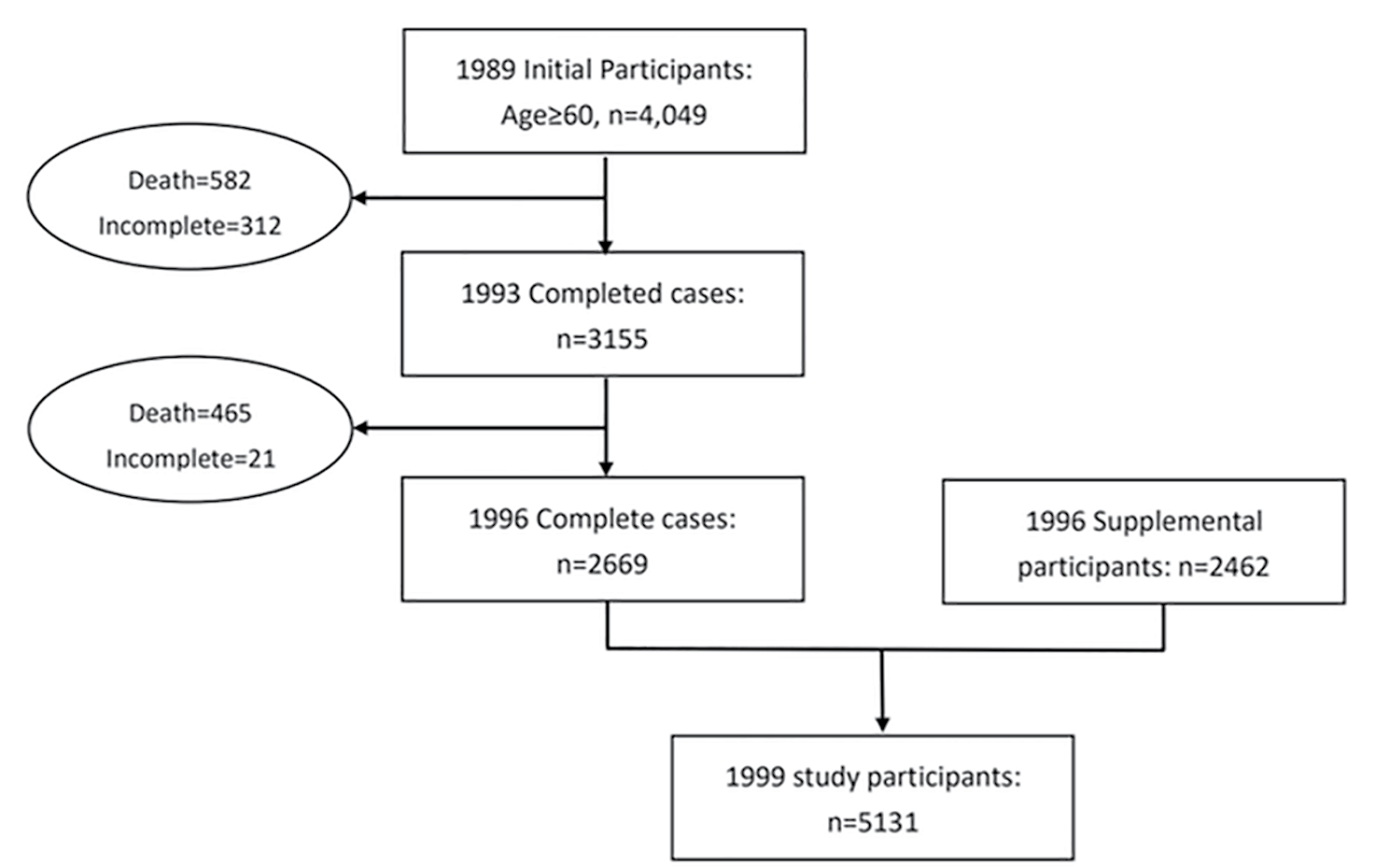
Flow chart of participants included in this study

All participants included in this study were part of the ongoing Taiwan Longitudinal Study of Aging (TLSA) initiated in 1989, with interviews conducted every 3–4 years [18]. The TLSA is a population-based prospective cohort study that was initiated by the Health Promotion Administration, Ministry of Health and Welfare, Taiwan. This survey was first conducted in 1989 among adult residents aged ≥ 60 years in the nonaboriginal townships of Taiwan with follow-ups conducted in 1993, 1996, 1999, 2003, 2007, 2011, and 2015 [19]. Details regarding the TLSA study design have been published [20–22]. The TLSA is a nationally representative population-based cohort study with a three-stage systematic random sampling design proportional to size sampling strategies. Participants were first recruited from 331 townships that were stratified by the administrative level, three levels of education, and three levels of the total fertility rate into 27 strata of roughly equal sizes. Subsequently, systemic random sampling was performed using the interval of selection equal to the size of each selected township divided by the number of blocks. Data were collected through face-to-face personal interviews conducted by trained interviewers. Two fresh population samples were selected by the TLSA study group in collaboration with the Population Studies Center, University of Michigan in 1996 and 2003, respectively, to maintain the representativeness of the younger age cohort and to extend the representativeness of the sample to the population aged ≥ 60 years. In the 2007 survey, a total of 3727 participants completed interviews, resulting in a high response rate of 88.3%. The details and design of the TLSA were described elsewhere [23]. The initial cohort in 1989 included 4049 participants aged ≥ 60 years. Later, in 1996, additional 2462 participants aged 60–66 years were included in the original cohort. Therefore, the 1996 TLSA sample represented the entire Taiwanese population aged ≥ 60 years living in an institution or in the community (n = 5131). Accordingly, the data of 5131 participants were used in the current study. Figure 1 presents the flow chart of participants included in this study.

## Measurements

### Dependent variable: frailty

Frailty is a geriatric condition characterized by accumulated deficits in multiple interrelated systems and decreased physiological reserves [4]. We adopted Markel–Reid and Browne and Rockwood definitions to examine frailty in this study [24, 25]. Frailty is defined as a multidimensional concept consisting of six domains, namely disease status, sensory dysfunction, balance while walking, functional limitation, health risk behaviors, and self-perceived health [24, 25]. For the disease status domain, the participants were asked for the presence of the following diseases: arthritis, cataract, respiratory disorders, hypertension, heart disorders, diabetes, liver or gallbladder disorders, stomach or intestinal ulcer, and kidney disorders. A score of 1 was assigned if the participants reported the presence of a certain disease. For the sensory dysfunction domain, a score of 1 was assigned if the participants reported having one of the following dysfunctions: eyesight, hearing, and chewing ability with dentures. Furthermore, a score of 1 was assigned for the balance while walking domain if the participants reported having a problem while walking. The functional limitation domain consists of incontinence, instruments of ADL (IADLs), mobility tasks, and participation in social activities. IADLs includes various activities such as grocery shopping, managing money, riding a bus, performing heavy and light housework, and using the telephone. In terms of mobility tasks, the participants’ ability to stand for 15 min, squat, lift their arms overhead, grasp with fingers, lift 12 kg, jog 20 to 30 m, climb stairs, and walk 200 to 300 m was evaluated. The degree of participation in social activities was examined based on the participants’ response to watching TV, reading newspapers, playing games, conversing, gardening, or participating in group activities including participating in religious organizations, worker unions, political parties, social services, and other social groups. Participants were assigned a score of 1 point if they reported difficulty in conducting the aforementioned items. To assess health risk behavior, which is the fifth domain, the participants were asked regarding their smoking, drinking, and betelnut-chewing behaviors. The sixth dimension consists of self-perceived health, life satisfaction, and depression. The current health condition and a comparison with previous year’s health condition were treated as self-perceived health. To evaluate life satisfaction, the participants were asked questions examining their attitude toward life, including “Are you satisfied with your life?” “Can your life be better than now?” “Are these the best years of your life?” and “Has most of your life been boring?”. The maximum summed score was 66. Cronbach’s α for the overall score ranged from 0.68 (1998) to 0.80 (2007) [26].

### Independent variable: physical activity trajectory

The physical activity frequency was evaluated using the following question: “How often do you usually exercise”? The response options were “no”, “≤2 times/week,” “3–5 times/week,” and “≥6 times/week” for group-based trajectory models. To determine the optimal number of groups that best describe our data, three approaches were used. The first approach was the use of the Bayesian information criterion (BIC). A more favorable BIC was the one with a smaller absolute value. The second approach was to ensure that the minimum group size for each group was in the range of 5% of the study population for interpreting the association of the groups with outcomes [27]. The third approach was to examine whether the trajectories were interpretable.

### Control variables

#### Demographic variables and comorbidities

From 1999, we collected data for variables that were considered risk factors for frailty in previous studies (i.e., age, sex, education level, income, smoking, and drinking). The education level was classified into four groups: 0, 1–6, 7–12, and > 12 years. The income level was determined by asking the following question: “Are you satisfied with your income?” The participants were allowed to answer to one of these three responses: good (very satisfied/satisfied), fair, and poor (unsatisfied and very unsatisfied). Chronic kidney disease and DM were suggested to be associated with frailty [28]. Thus, kidney disease and DM were considered as risk factors for frailty.

#### Oral health

Oral health was found to be associated with cognitive ability and frailty [28]. The Oral Health Impact Profile (OHIP) is the most commonly used instrument for evaluating oral disorders [29]. The model of oral health–related quality of life (OHRQoL) hypothesizes that oral conditions and symptoms may be positively related to functional limitations and thus physical pain and psychological discomfort [30]. In addition, a study reported that oral pain, chewing problems, and decreased masticatory function have been associated with being either frail [31] or having lower cognitive function [32] in older adults. Thus, we adopted two items from the OHIP to measure oral disorders: “OHIP-7T Q3: uncomfortable to eat” and “OHIP-7T Q7: worse taste” [28]. Each item is rated from 0 (never) to 4 (very often) [27], rendering a total score of 0–8, with higher score denoting worse OHRQoL.

#### Well-being index

To examine the well-being of the participants, the World Health Organization-Five Well-Being Index (WHO-5) used to measure mental status in both somatic and psychiatric disorders was adopted. The WHO-5 was validated to have satisfactory psychometric properties and correlation with a depression rating scale [33]. The participants were asked how they had felt over the last 2 weeks and allowed to choose from the following responses: “I have felt cheerful and in good spirits,” “I have felt calm and relaxed,” “I have felt active and vigorous,” “I woke up feeling fresh and rested,” and “My daily life has been filled with things that interest me.” Each item is rated from 0 (not at all) to 5 (all the time), given a summary score ranging from 0 to 25 [33]. A higher score indicates the better well-being.

#### Depressive symptoms

A shorter version of the Center for Epidemiologic Studies Depression Scale (CESD) was adopted to measure depressive symptoms [34]. The CESD contains 10 items rated on a scale of 0 to 3. CESD asks participants to rate how often over the past week they experienced symptoms associated with depression, such as restless sleep, poor appetite, and feeling lonely. The suggested cut-off point for the detection of depression is 16 [35, 36].

#### Ethics

Informed consent obtained from the study participants prior to TLSA study commencement. The permission of accessing the TLSA study database was granted by the Ministry of Health and Welfare in Taiwan. The study protocol of this longitudinal analysis was reviewed and approved by Taipei Medical University Joint Institutional Review Board, approval number: N201906072.

### Statistical analysis

In the current study, each hypothesis was tested using two-sided alternatives with a significance level of 0.05. The standardized mean difference was calculated to determine differences between the groups. Differences between the groups were examined using the independent sample t test for continuous variables and the *χ*^2^ test for categorical variables.

Information regarding the duration of physical activity retrieved from the TLSA at year 1996, 1999, 2003, 2007, 2011, and 2015 was analyzed using group-based trajectory modeling (GBTM) to classify homogeneous trajectory clusters. GBTM is a special case of finite mixture models that provides the empirical foundation for longitudinal analysis by classifying subgroups that demonstrate exercising trajectories. The model classifies individuals into clusters with similar trajectories [37] according to the longitudinal data of each individual, assuming that individual differences in trajectories can be summarized by different polynomial functions for time [38]. The BIC was used to measure the goodness of fit for the results obtained from GBTM. After the identification of potential physical activity trajectories, potential predictors were examined by performing logistic regression analysis to determine significant predictors for frailty. Using the group without frailty as the reference category, we calculated adjusted odds ratios with 95% confidence intervals (CIs) to investigate the effects of significant predictors on the physical activity category. All analyses were performed using SAS version 9.4. In particular, the *Proc Traj macro* was adopted to perform GBTM.

## Results

### Demographics and baseline information of study participants

Table 1 lists the demographic information of the collected sample. The sample consisted of 5131 patients enrolled from 1996 to 2015. The proportion of men (53.79%) was slightly higher than that of women (46.21%). The average age of the participants was 66.21 (standard deviation: 9.42) years. Of the total participants, 24.3% and 7.7% had DM and chronic kidney disease, respectively. Approximately 48.9% of the participants did not engage in physical activity, and 38.1% of the participants exercised 7 times a week. Furthermore, 19.2% and 18.8% of the participants were aged between 70 and 74 years and between 65 and 69 years, respectively. Approximately 5.6% of the participants reported mental health problems.

**Table 1:** Baseline information of study participants

	Male (n = 2760)		Female (n = 2131)		Overall	
Physical activity frequency (per week, %)						
0	1249	45.3	1257	53	2506	48.9
2	144	5.2	131	5.5	275	5.4
4	204	7.4	191	8.1	395	7.7
7	1162	42.1	791	33.4	1953	38.1
Age (mean [SD])	66.34	9.16	66.06	9.71	66.22	9.40
Education (years, %)						
0	415	15	1258	53.1	1673	32.6
1–6	1253	45.4	756	31.9	2009	39.2
7–9	341	12.4	134	5.7	475	9.3
10–12	294	10.7	70	3	364	7.1
13–17	248	9	34	1.4	282	5.5
SES (mean [SD])	4.81	1.91	4.62	1.93	4.71	1.92
Diabetes mellitus (%)	189	22.4	239	26.1	428	24.3
Chronic kidney disease (%)	68	8.1	67	7.3	135	7.7
OHIPq3 (mean [SD])	0.46	0.5	0.53	0.5	0.49	0.5
OHIPq7 (mean (SD))	0.25	0.43	0.26	0.44	0.26	0.44
Hypertension (%)	426	97.3	544	97.3	970	97.3
CVD (%)	188	92.2	253	94.8	441	93.6
Stroke (%)	92	10.9	104	11.4	196	11.1
Mental health (%)	35	4.2	64	7	99	5.6
Bone fracture (%)	26	3.1	64	7	90	5.1
Sleep quality (mean [SD])	2.03	0.81	2.34	0.85	2.19	0.85
Smoke (%)	137	27	10	45.5	147	27.8
Drink (%)	245	29.1	48	5.2	293	16.7
SPMSQ (mean [SD])	9.97	1.67	8.94	2.45	9.44	2.17
Loneliness (mean [SD])	6.6	2.03	6.36	1.85	6.48	1.94
Life satisfaction (mean [SD])	3.22	1.27	3.04	1.39	3.13	1.33
Balance dysfunction (mean [SD])	0.71	0.45	0.57	0.49	0.64	0.48
IADL (mean [SD])	1.85	2.8	2.66	3.02	2.27	2.94
ADL (mean [SD])	3.02	3.18	4.41	3.19	3.74	3.26
CESD (mean [SD])	7.8	4.1	9.32	4.72	8.58	4.49
Social activities (mean [SD])	12.33	1.97	12.51	1.76	12.42	1.87
Health risk behavior (mean [SD])	0.55	0.72	0.08	0.31	0.31	0.6
Perceived health (mean [SD])	0.65	0.48	0.55	0.5	0.6	0.49

The percentage was based on the corresponding responses divided by the total number of responders. SES = self-reported social economic status. OHIPq3 = Oral Health Impact Profile question 3: Are you uncomfortable to eat due to an oral problem in recent 12 months? OHIPq7 = Oral Health Impact Profile question 7: Do you experience a worse taste due to an oral problem in recent 12 months? CVD = cardiovascular disease. SPMSQ = Short Portable Mental Status Questionnaire. IADL = Instrumental Activities of Daily Living. ADL = Activities of Daily Living. CESD = Center for Epidemiologic Studies Depression Scale

### Physical activity trajectory

**Fig. 2:**
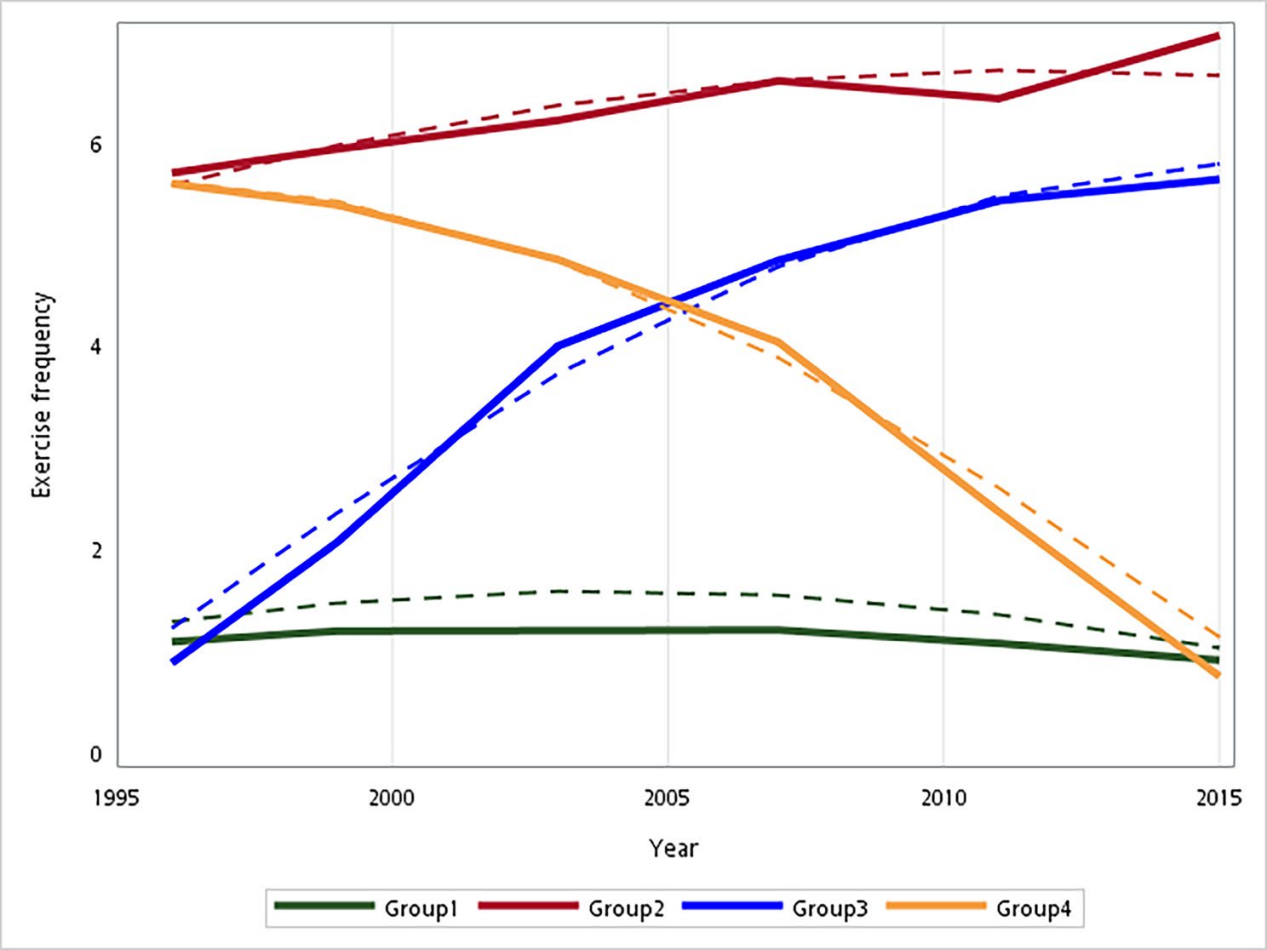
Physical activity trajectories of being consistently physically inactive (group 1), consistently physically active (group 2), incline (group 3), and Decline (group 4)

According to the criteria of the smaller BIC, minimum group size, and interpretability, we selected the four-group model for further analysis. To illustrate the trajectories of the physical activity pattern in the GBTM framework, we plotted the predicted mean trajectory of physical activity frequency over 20 years (Fig. 2). As shown in Fig. 2, we identified four trajectories of the physical activity pattern: consistently physically inactive (33.7%), consistently physically active (21.5%), incline (21.6%), and decline (23.2%). Table 2 presents a comparison of baseline characteristics. Table 3 shows the results of the multivariate logistic regression model.

**Table 2:** Comparisons of physical activity patterns for frailty and its subdomains measured at 2015

	Physical activity pattern								
	Consistently inactive (n = 2001)		Consistently active (n = 1077)		Incline (n = 785)		Decline (n = 1268)		p
Age (mean [SD])	66.5	9.24	64.78	7.85	60.92	7.69	67.49	7.63	< 0.001
Female (%)	1039	51.9	368	34.2	375	47.8	589	46.5	< 0.001
OHIPq3 (mean [SD])	0.56	0.5	0.42	0.49	0.48	0.5	0.52	0.5	0.001
OHIPq7 (mean [SD])	0.29	0.46	0.22	0.41	0.25	0.43	0.28	0.45	0.097
SPMSQ (mean [SD])	9.42	2.14	9.82	1.91	9.51	2.04	8.78	2.64	< 0.001
Loneliness (mean [SD])	6.45	2.09	6.56	1.97	6.41	1.8	6.49	1.89	0.744
Life satisfaction (mean [SD])	2.89	1.43	3.37	1.21	3.24	1.26	2.93	1.4	< 0.001
CESD (mean [SD])	9.21	5.06	8.07	3.74	8.07	4.09	9.29	5.1	< 0.001
Frailty score at 2015 (mean [SD])	26	6.36	23.34	5.36	23.49	5.8	28.26	6.96	< 0.001
Frailty subdomain – Disease status (mean [SD])	1.77	1.39	1.8	1.27	1.78	1.25	2.21	1.46	< 0.001
Frailty subdomain – Sensory dysfunction (mean [SD])	0.5	0.7	0.32	0.55	0.35	0.62	0.58	0.73	< 0.001
Frailty subdomain – Balance dysfunction (mean [SD])	0.63	0.48	0.72	0.45	0.76	0.43	0.41	0.49	< 0.001
Frailty subdomain – Functional limitation measured by IADL (mean [SD])	2.64	3.15	1.31	2.1	1.41	2.32	4	3.32	< 0.001
Frailty subdomain – Functional limitation measured by ADL (mean [SD])	4.14	3.41	2.7	2.64	2.8	2.78	5.61	3.34	< 0.001
Frailty subdomain – Functional limitation measured by Social activities (mean [SD])	12.94	1.6	11.94	2.02	11.84	1.85	13.05	1.62	< 0.001
Frailty subdomain – Health risk behavior (mean [SD])	0.33	0.62	0.32	0.6	0.36	0.65	0.2	0.46	< 0.001
Frailty subdomain – Perceived health (mean [SD])	0.59	0.49	0.68	0.47	0.61	0.49	0.48	0.5	< 0.001

The percentage was based on the corresponding responses divided by the total number of respondents. OHIPq3 = Oral Health Impact Profile question 3: Are you uncomfortable to eat due to an oral problem in recent 12 months? OHIPq7 = Oral Health Impact Profile question 7: Do you experience a worse taste due to an oral problem in recent 12 months? SPMSQ = Portable Mental Status Questionnaire Short. CESD = Center for Epidemiologic Studies Depression Scale. IADL = Instrumental Activities of Daily Living. ADL = Activities of Daily Living

**Table 3:** Multivariate logistic regression for physical activity trajectories for frailty

Parameter	Beta	SE	P	OR	95% Wald confidence limits	
Age	0.7247	0.069	< 0.00001	2.064	1.803	2.363
Male (ref: Female)	−0.2881	0.0816	0.0004	0.562	0.408	0.774
DM (ref: No)	0.6883	0.1816	0.0002	1.99	1.394	2.841
Kidney disease (ref: No)	1.233	0.2702	< 0.0001	3.432	2.021	5.828
OHIPq3	0.46	0.1814	0.0112	1.584	1.11	2.26
OHIPq7	0.3395	0.1866	0.0688	1.404	0.974	2.024
CESD	1.0923	0.2335	< 0.0001	2.981	1.886	4.712
Life Satisfaction	−0.5763	0.0595	< 0.0001	0.562	0.5	0.631
Baseline Frailty	2.2359	0.6753	0.0009	9.355	2.49	35.142
Consistently active group (ref: Consistently inactive group)	−1.0023	0.2291	< 0.0001	0.367	0.234	0.575
Incline group (ref: Consistently inactive group)	−0.7963	0.2177	0.0003	0.451	0.294	0.691
Decline group (ref: Consistently inactive group)	−0.065	0.2244	0.772	0.937	0.604	1.455

DM = diabetes mellitus. OHIPq3 = Oral Health Impact Profile question 3: Are you uncomfortable to eat due to an oral problem in recent 12 months? OHIPq7 = Oral Health Impact Profile question 7: Do you experience a worse taste due to an oral problem in recent 12 months? CESD = Center for Epidemiologic Studies Depression Scale. Consistently active group = consistently active in physical activity trajectories. Incline group = incline in physical activity trajectories. Decline group = decline in physical activity trajectories

Across the study period, the consistently physically active group maintained frequent physical activity, whereas the incline group showed a quadratic increase in the physical activity pattern, the decline group exhibited a nonlinearly decreasing physical activity pattern and the consistently physically inactive group maintained minimum physical activity. These four trajectory groups started at a considerably different intercept at the beginning of the study. The consistently decline group was similar to the decline group, whereas the consistently incline group was similar to the incline group. By 2015, the declining group engaged in less physical activity, whereas the inclining group continued to increase their physical activity frequency (Fig. 2). Throughout the period, the trajectories of the four groups significantly differed from each other at year 2015, with the incline and decline groups exhibiting the lowest and highest frailty scores, respectively (p < 0.001; Table 2). Among the six subdomains of frailty, the incline group outperformed the other three groups in disease status (1.78 ± 1.25) and social activities (11.84 ± 1.85), whereas the decline group exhibited the poorest performance in disease status (2.21 ± 1.46), sensory dysfunction (0.58 ± 00.73), IADL (4 ± 3.32), ADL (5.61 ± 3.34), and social activities (13.05 ± 1.62). In addition to frailty, the incline group experienced the least loneliness (6.41 ± 1.8) and the highest life satisfaction (3.24 ± 1.26). The declining and consistently physically inactive groups both exhibited lower cognitive function in terms of the Short Portable Mental Status Questionnaire (SPMSQ) (8.78 ± 2.64 and 9.42 ± 2.14, respectively) and higher depressive symptoms, as determined using the CESD (9.29 ± 5.1 and 9.21 ± 5.06, respectively).

### Predicting outcomes of frailty by using physical activity trajectories

To investigate the effect of the physical activity trajectories from 1996 to 2015 on the frailty status at year 2015, we conducted multivariate logistic regression. The frailty status was defined as a binary variable by using a cutoff point of 30. The optimal cutoff point was determined using the area under the receiver operating characteristic curve of frailty in relation to death with the Youden index. Age, male sex, DM, chronic kidney disease, oral health, and high CESD scores which were measured at baseline were identified as risk factors for frailty, whereas life satisfaction was determined as a protective factor for frailty (all p < 0.05, Table 3). After adjustment for potential covariates, different physical activity patterns could still significantly explain the probability of being frail. Compared with the consistently physically inactive group, the incline group had a 54.9% lower risk of frailty (OR = 0.451, 95% CI = 0.294–0.691). Furthermore, the consistently physically active group had a 63.3% lower risk of frailty than did the consistently physically inactive group. The decreased probability of being frail could be as low as 76.6% if a participant engaged in physical activity behavior (OR = 0.637, 95% CI = 0.234–0.575).

**Fig. 3:**
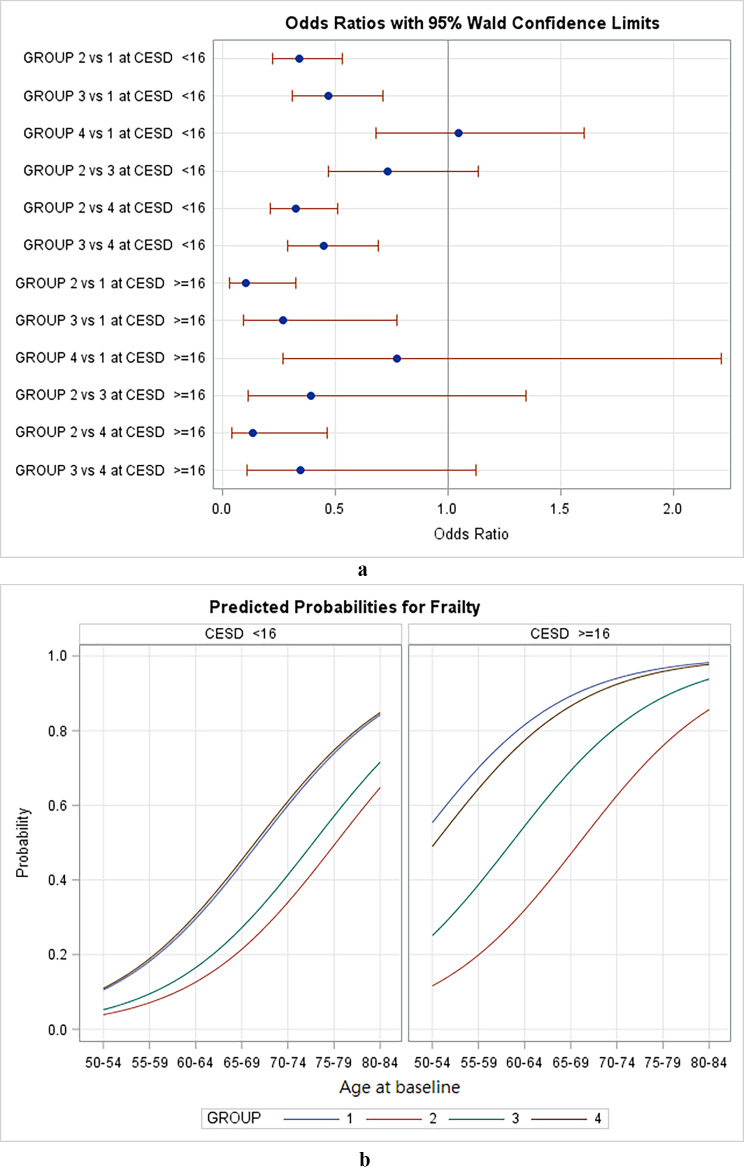
Effect of physical activity trajectories on frailty stratified by depression status (a) predicted probabilities for frailty between groups; (b) comparison of odds ratios and 95% Wald confidence interval between groups

A strong correlation was observed between frailty and depressive symptoms. Thus, we conducted sensitivity analysis to investigate the effects of the physical activity patterns on frailty under different levels of depressive symptoms (Fig. 3). The risk of frailty in the four physical activity trajectories was higher in the participants who exhibited depressive symptoms. That is, the risk of frailty in the four physical activity trajectories was lower in the participants who did not show depressive symptoms than in those who exhibited depressive symptoms (Fig. 3a). The incline group had a significantly lower risk of frailty than did the consistently physically inactive group regardless of the presence of depressive symptoms (Fig. 3b). The consistently physically inactive group had a higher risk of frailty than did the consistently physically active and incline groups in both the participants with and without depression.

## Discussion

### Benefits of maintaining or increasing physical activity

Physical activity and regular exercise are interventions frequently considered to contribute to healthy ageing and that older people participate in. The extent of participation and engagement of older people in physical activities highly depend on the context and physical demands of a particular activity [7, 19, 39]. Our longitudinal data indicated that physical activity is associated with a reduced risk of frailty status in the older people who maintained and increased physical activity as they age. Although regular exercise and physical activity have numerous health benefits, pointing out such benefits in the older adult population is challenging because of the varying levels of overall health status and normal age-related physiological changes resulting in functional limitations. Our study results indicated that instead of primarily focusing on health benefits in older people, an observation of the longitudinal trend and frailty risk can provide a more realistic understanding of the benefits of regular exercise and physical activity in advancing age.

The older adults in our study who increased their physical activity levels despite experiencing health problems were observed to be less frail. Older people who remained physically active in their 80s were less likely to be hospitalized compared with those who were less physically active [40, 41]. The activity and physical activity patterns of the older people in our study had four trajectories. The older people who were consistently physically active and those who increased their physical activity levels were observed to be less frail. Low frailty scores reflected better cognitive function, OHRQoL, and positive perception toward life. Although studies have identified these factors as individual frailty measures [2, 31, 42, 43], they have not been examined together with physical activity trajectories. We observed that the depression scores were lower in the older people with a low risk of frailty. Our findings suggested that physical activity should be encouraged in older people and increasing the physical activity level can reduce the risk of frailty. Identifying the patterns and threshold levels of increased physical activity might be crucial in the consideration of health status and presence of comorbidities.

### Predicting outcomes of frailty by using physical activity trajectories

Using physical activity trajectories for examining the risk of frailty in the older Taiwanese people, we identified that two chronic conditions, namely DM and kidney disease, strongly predicted the risk of frailty. This finding is in accordance with those of previous studies. For example, in their systematic review, Hanlon et al. (2020) found that frailty was associated with hypoglycemia, severe complications, and poor quality of life in patients with diabetes [42]. In addition, patients with chronic kidney disease, particularly those undergoing dialysis, had frailty and poor health [44]. However, in terms of physical activity patterns, these conditions usually lead to limited functional and physical abilities, and these diseases can affect cognitive functioning with their progression (i.e., increased levels of creatinine and ketones in the blood). Thus, these diseases might have contributed to frailty in the older people examined in this study.

In terms of the presence of chronic conditions, our results revealed oral health as a risk factor for frailty. Satisfactory oral health is linked to nutritional intake, which, in turn, will have a knock-on effect on overall health status in old age. In a systematic review of longitudinal studies conducted in the United Kingdom, Japan, and Mexico, a significant association was observed between oral health and frailty. Furthermore, this study suggested that mediating factors such as nutrition should be examined in older people [45]. Our findings support the results of these earlier studies and highlight that diseases such as DM and chronic kidney disease that present with metabolic deficiencies might have some link with nutritional problems associated with frailty and oral health in older adults.

A study examined the association between depression and overall outlook in life and found that depressive symptoms were associated with frailty [46]. Supporting our findings regarding depression and frailty, a previous study reported a high prevalence of depressive disorders in a sample of community-dwelling older people in Taiwan enrolled between the mid-1996 and mid-1998 [47]. The extent of and actual mechanism through which depression in older Taiwanese people predicts frailty, particularly when interpreted in those who are consistently physically inactive, should be elucidated. Although positive outlook or satisfaction in life is a protective factor for frailty, these measures can be highly subjective and depend on personal expectations. Satisfaction in life mediates coping measures and depressive symptoms, which were more pronounced in older age groups [48, 49].

### Strengths and limitations

This study has several strengths. This study used nationally representative longitudinal data for over 20 years to investigate physical activity trajectories in relation to frailty in the older people. In addition, this study had a robust retrospective design with secondary analysis and included a large sample size. A limitation of this study is that confounding variables might have been limited; thus, all possible confounders could not be included due to the limitation of secondary analysis. Nevertheless, we performed a comprehensive literature review to identify possible control variables and used a rigorous statistical approach in the current study. Physical activity was defined as any bodily movement that requires energy expenditure. However, in this retrospective design, we do not have detailed information regarding the types of physical activity that linked to the frequency of the reported physical activity pattern. As a result, the association of different physical activity type on onset of frailty was not investigated in current study. A further limitation is that correlation analyses were performed with retrospective design and thus no cause-and-effect statements can be derived. Cluster-randomized controlled intervention studies are necessary to investigate the effects of physical activity on frailty and comorbidities in older adults.

We highlighted potential risk factors including age, gender, diabetes mellitus, oral health, depression, life satisfaction, and physical activity pattern in the prediction of frailty. The results of this study indicated that compared with older people who exercised less or reduced their physical activity, those who were active had a lower risk of frailty. Compared with people who were consistently physically inactive, older adults who exercised consistently had a much lower risk of being frail. This protective effect was similar to the results for people who began exercising regularly in their 60s. It is never be too late to start exercising. Since we have demonstrated the composite risk factor for frailty, a frailty risk assessment is applicable and the findings of the current study are crucial to guide the development of future research and interventions to screen and manage frailty in older people.

## Data Availability

The data that support the findings of this study are available from Ministry of Health and Welfare in Taiwan but restrictions apply to the availability of these data, which were used under license for the current study, and so are not publicly available. Data are however available from the authors upon reasonable request and with permission of Ministry of Health and Welfare in Taiwan.

## List of abbreviations

TLSA: Taiwan Longitudinal Study of Aging
SES: self-reported social economic status
OHIPq3: Oral Health Impact Profile question 3: Are you uncomfortable to eat due to an oral problem in recent 12 months?
OHIPq7: Oral Health Impact Profile question 7: Do you experience a worse taste due to an oral problem in recent 12 months?
CVD: cardiovascular disease
SPMSQ: Short Portable Mental Status Questionnaire
IADL: Instrumental Activities of Daily Living
ADL: Activities of Daily Living
CESD: Center for Epidemiologic Studies Depression Scale
OHRQoL: oral health–related quality of life
GBTM: group-based trajectory modelling
WHO: World Health Organization
DM: diabetes mellitus
BIC: Bayesian information criterion
